# Rewiring macrophage immunometabolism in gouty arthritis: from metabolic checkpoints to intelligent nano-delivery

**DOI:** 10.3389/fphar.2026.1794487

**Published:** 2026-04-29

**Authors:** Feng Xiao, Xin Zhou, Jie Liu, Kaijun Huang, Qixiang Qin, Chengxue Li, Yifan Lu, Hui Xiong, Xiaobin Luo, Zhiqiang Luo, Aitian Zhang, Wenjie Su

**Affiliations:** 1 Department of Orthopedics, Shaoyang Hospital of Traditional Chinese Medicine, Shaoyang, Hunan, China; 2 The First Clinical Medical College of Heilongjiang University of Chinese Medicine, Harbin City, Heilongjiang, China; 3 Department of Orthopedics, Yongzhou Hospital of Traditional Chinese Medicine, Yongzhou, Hunan, China; 4 Hunan University of Chinese Medicine, Changsha, Hunan, China; 5 Department of Orthopedics, Longhui County Hospital of Traditional Chinese Medicine, Shaoyang, Hunan, China; 6 Department of Orthopedics, Xiangtan Hospital of Traditional Chinese Medicine, Xiangtan, Hunan, China

**Keywords:** glycolysis, gouty arthritis, immunometabolism, intelligent nano-delivery, lactylation, metabolic reprogramming, OXPHOS

## Abstract

Gouty arthritis (GA) is driven by NLRP3 inflammasome activation, yet its underlying metabolic mechanisms remain poorly explored. Current therapies focus on uric acid reduction and anti-inflammation, often overlooking the plasticity of macrophages controlled by metabolic reprogramming. This review systematically dissects the metabolic shifts in GA, particularly the transition from oxidative phosphorylation (OXPHOS) to glycolysis (Warburg effect) and the unique role of lipid/amino acid metabolism. We describe macrophage immunometabolism, tricarboxylic acid (TCA)cycle breakpoints, and metabolite functions, with particular emphasis on the “metabolic-epigenetic” axis (e.g., lactate conversion). We summarize emerging nanotherapeutic strategies (e.g., nanoenzymes, biomimetic carriers) precisely targeting these metabolic checkpoints. Current reviews on GA primarily focus on conventional anti-inflammatory and uric acid-lowering strategies. However, although macrophages are central drivers of the GA disease process, the mechanisms underlying the coupling of their metabolic reprogramming and polarization have not yet been fully elucidated. In particular, metabolite-mediated “metabolic-epigenetic” crosstalk, as well as how to precisely regulate these metabolic targets using emerging nanotargeting technologies, remain blind spots in current research. This paper is the first to systematically integrate these dimensions, aiming to fill this gap by exploring novel nanostrategies and future prospects for treating GA through the remodeling of macrophage immunometabolism. Targeting macrophage metabolism offers a paradigm shift for GA—from conventional symptom management to targeted disease resolution by directly inhibiting glycolytic flux and succinate accumulation, thereby repolarizing pro-inflammatory M1 macrophages into the tissue-repairing M2 phenotype.

## 1 Introduction

Acute gouty arthritis (AGA) is characterized by a rapid inflammatory response triggered by the accumulation of monosodium urate (MSU) crystals in periarticular tissues. This condition is typically associated with hyperuricemia (HUA) and presents with symptoms of severe pain, active inflammation, and joint swelling (Wang et al., 2024). As the most prevalent form of inflammatory arthritis, the global prevalence and incidence of GA continue to rise (Dehlin et al., 2020).

The inflammatory response induced by gout is characterized by the infiltration of multiple immune cells, with neutrophils and macrophages being particularly prominent (Zhao J. et al., 2022). Macrophages are considered one of the primary cells initiating and driving inflammation triggered by MSU crystals (Martin et al., 2009). Following recognition and phagocytosis of MSU crystals, macrophages rapidly activate the NLRP3 inflammasome (nucleotide-binding domain-like receptor family pyrin domain 3). This activation promptly triggers the release of the inflammatory protease caspase-1, thereby initiating a downstream inflammatory cascade (So and Martinon, 2017). Macrophages play a pivotal role in initiating acute gouty inflammation and the subsequent inflammatory cascade. However, recent studies indicate that the role of macrophages in gout is complex and multifaceted, extending beyond merely promoting harmful inflammatory effects. This is because distinct macrophage subtypes exhibit heterogeneous functional properties, each playing unique regulatory roles in the progression of gout and the resolution of inflammation (Tan et al., 2024a).

In recent years, metabolic therapy has emerged as a research hotspot due to its ability to regulate the metabolic profiles of immune cells for treating various diseases. Unlike traditional broad-spectrum immunosuppression, this emerging strategy aims to achieve selective and precise modulation of immune responses (Patel et al., 2019). This innovative approach is rooted in the recognition that immune cell activation and differentiation are closely linked to metabolic reprogramming, a phenomenon first described in cancer cells as the Warburg effect, characterized by increased glycolysis and reduced OXPHOS (Fresquet et al., 2021). M1 macrophages are primarily glycolytically powered, with impaired TCA cycle and mitochondrial OXPHOS processes. Conversely, M2 macrophages rely predominantly on OXPHOS for energy acquisition. Altering the metabolic profile of macrophages can modify their phenotype (Ao-Di et al., 2024). Macrophages play a central role in the initiation and resolution of inflammation in GA. MSU crystals can regulate the M1/M2 polarization of macrophages by activating multiple signaling pathways, prompting them to release various inflammatory mediators—including pro-inflammatory cytokines and chemokines—either independently or in concert, ultimately mediating and driving the inflammatory response (Zhao et al., 2024). In particular, the infiltration of M1-type macrophages into the joint has been identified as a key factor exacerbating the disease, and the extent of this infiltration is highly positively correlated with the clinical severity of arthritis (Song et al., 2026). In exploring the underlying mechanisms, it is widely recognized in the academic community that metabolic reprogramming of macrophages profoundly influences the onset, progression, and resolution of the aforementioned inflammation. The pathological progression of GA is accompanied by complex feedback mechanisms: on the one hand, metabolic abnormalities in the local joint microenvironment drive dynamic changes in macrophage energy metabolism; on the other hand, metabolic reprogramming of macrophages also acts back on the microenvironment, exacerbating local metabolic dysregulation (Ahn and So, 2025). Existing evidence suggests that metabolic regulation is the cornerstone driving macrophage phenotypic conversion. Specifically, pro-inflammatory M1 macrophages primarily rely on glycolysis for energy supply; whereas immunosuppressive M2 macrophages mainly depend on fatty acid oxidation (FAO) and OXPHOS to meet their metabolic demands (Ma et al., 2025). Based on the aforementioned mechanisms, the key to breaking this vicious cycle lies in reprogramming the inflammatory immune microenvironment. This is primarily manifested by the downregulation of pro-inflammatory cytokines and ROS levels, increased expression of anti-inflammatory cytokines, and the polarization of pro-inflammatory M1 macrophages into anti-inflammatory M2 macrophages, thereby providing important therapeutic targets for the treatment of GA (Chen et al., 2023). Given these findings, regulating the energy metabolism of macrophages in GA to intervene in their polarization state and thereby reduce inflammation represents a key future research direction.

Furthermore, metabolic reprogramming in macrophages enhances glycolysis and promotes excessive lactate accumulation (Xu B. et al., 2024). Lactate has traditionally been regarded as both an energy substrate and metabolic waste product. However, recent evidence has repositioned it as a critical signaling molecule. Further studies reveal that lactate-derived histone lysine residue lactylation represents a novel epigenetic modification directly driving gene transcription on chromatin (Zhang et al., 2019). Lactate-dependent histone modification constitutes a novel histone tag linking glycolytic metabolites to lactylated epigenetic processes (Li et al., 2024). Thus participating in the pathogenesis of multiple diseases, including GA.

**FIGURE 1:**
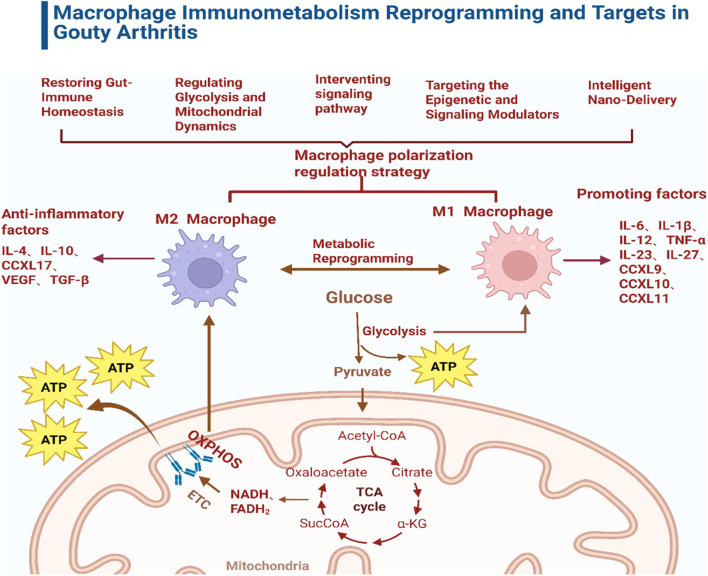
Macrophage immunometabolism reprogramming and targets in gouty arthritis.

The role of macrophage metabolic reprogramming in GA inflammation has become a current research hotspot, with the “metabolic-epigenetic” axis—represented by lactonization modification—emerging as a Frontier direction. This paper systematically reviews the mechanisms of macrophage metabolic reprogramming and the physiological functions of its immunometabolites, focusing on the “metabolic-epigenetic” regulatory network in the context of GA. It also thoroughly explores therapeutic strategies targeting macrophage polarization and metabolism (see Figure 1).

Although several excellent recent reviews have comprehensively described the overall pathogenesis of gout, these reviews have primarily focused on the deposition of MSU crystals and the activation of the classical NLRP3 inflammasome. However, the complex metabolic reprogramming that determines the fate of immune cells—particularly the dynamic polarization of macrophages in the gout microenvironment—remains a relatively fragmented area in the current literature. Unlike previous broad overviews, this article systematically elucidates the critical role of macrophage metabolic reprogramming in GA. We clearly demonstrate how the shift toward aerobic glycolysis and the accumulation of TCA cycle intermediates—such as succinate and citrate—drive pro-inflammatory M1 polarization. More importantly, this review represents the first interdisciplinary integration in the field of gout inflammation between the underlying mechanisms of “macrophage metabolic reprogramming-driven phenotypic polarization” and cutting-edge “smart nano-targeted delivery systems.” This approach aims to provide a novel perspective on elucidating the inflammatory progression of GA and lays the theoretical foundation for the future development of a new generation of precision therapies based on metabolic intervention and epigenetic regulation.

## 2 Basic concepts of macrophage metabolic reprogramming

As the body’s homeostasis shifts and relevant pathological products and exogenous substances infiltrate, macrophages can activate associated metabolic patterns to adapt to these changes. This environmentally adaptive metabolic shift is termed metabolic reprogramming of macrophages (Sun et al., 2022). Macrophages are highly plastic cells, and macrophage polarization is the process by which they respond to stimuli from the local microenvironment and acquire specific functional phenotypes. Macrophages are typically categorized into classically activated, pro-inflammatory, or M1 macrophages, and alternatively activated, anti-inflammatory, or M2 macrophages (Viola et al., 2019). Metabolism influences the microenvironment, primarily through alterations in metabolic products. Similarly, metabolism serves as a crucial regulator of innate immunity and inflammation, with macrophages exhibiting high sensitivity to metabolic alterations (Pesce et al., 2009; Rodríguez-Prados et al., 2010). Thus, metabolism modulates the functional responses of macrophages, and the ability to detect metabolic changes in macrophages is crucial for maintaining homeostasis in the microenvironment (Shalova et al., 2015). Current metabolic reprogramming in macrophages primarily involves glycolysis, the TCA cycle and oxidative phosphorylation, fatty acid metabolism, and amino acid metabolism.

### 2.1 Glycolysis

Glycolysis is a crucial pathway by which organisms generate energy by converting glucose into ATP without using oxygen. It serves as the common starting point for both anaerobic and aerobic glucose oxidation, producing byproducts such as lactate, pyruvate, and adenosine diphosphate (ADP) (Ao-Di et al., 2024). Aerobic glycolysis, or the Warburg effect, is characterized by enhanced glucose uptake and lactate production (Feng et al., 2020). Key enzymes regulating glycolysis include hexokinase 2 (HK2), phosphofructokinase-1 (PFK1), and pyruvate kinase (PK) (Fernie et al., 2004).

Hexokinase (HK) is the first rate-limiting enzyme in aerobic glycolysis, catalyzing the conversion of glucose to glucose-6-phosphate (G-6-P) (Lis et al., 2016). The enzyme has four subtypes (HK1, HK2, HK3, and HK4), but most normal tissues express only HK1. HK2 is the most efficient enzyme promoting aerobic glycolysis among the HK isoforms (Xu and Herschman, 2019; Feng et al., 2020). HK2, as the key rate-limiting enzyme catalyzing the first step of glucose metabolism, plays a central role in driving cellular metabolic reprogramming. Extensive evidence indicates that even under oxygen-rich conditions, high expression of HK2 can significantly accelerate glucose metabolism, thereby inducing the classic Warburg effect (Li J. et al., 2025). In the immune microenvironment, HK2-mediated metabolic reprogramming is not only a hallmark of metabolic shifts in macrophages but also a key molecular switch that directly drives their polarization toward the pro-inflammatory M1 phenotype and triggers NLRP3 inflammasome activation (Tan et al., 2024b).

PFK1 is the second rate-limiting enzyme involved in glycolysis, catalyzing the conversion of fructose-6-phosphate (F-6-P) to fructose 1,6-bisphosphate (F-1,6-BP) using ATP (Kanai et al., 2019). The latter regulates glycolytic flux and determines cell fate. Emerging research indicates that there are three tissue-specific PFK-1 isoforms: the platelet isoform (PFKP), the liver isoform (PFKL), and the muscle isoform (PFKM) (Yuan et al., 2025). During the activation phase of macrophages, the TGF-β signaling axis significantly upregulates the expression and catalytic activity of PFKL, a key enzyme in glycolysis, thereby accelerating the overall glycolytic metabolic flux (Gauthier et al., 2023). This PFKL-mediated reprogramming of basal metabolism directly drives the reversal of macrophages from the anti-inflammatory M2 phenotype to the pro-inflammatory M1 phenotype (Dang et al., 2023).

The final rate-limiting enzyme in glycolysis is PK, which catalyzes the conversion of PEP into ATP and pyruvate. PK also has four isoforms, including hepatic PK (PKL), erythrocyte PK (PKR), and the muscle PK isoenzymes M1 and M2 (PKM1 and PKM2, respectively) (van Niekerk and Engelbrecht, 2018).

Pyruvate kinase M2(PKM2) exists in two forms: a dimer with lower pyruvate kinase activity and a tetramer with higher pyruvate kinase activity. These forms can interconvert through modifications such as dephosphorylation/phosphorylation and deacetylation/acetylation. This conformational switching in PKM2 serves as a critical switch in glycolysis, catalyzing the breakdown of glycolytic intermediates to ultimately produce ATP. It represents a pivotal metabolic step in the Warburg effect (aerobic glycolysis) (Xu et al., 2019; Zahra et al., 2020; Zheng et al., 2021). PKM2 is a key target mediating metabolic adaptation in macrophages. Studies have shown that lactate can induce anti-inflammatory polarization of macrophages from the M1 phenotype to the M2 phenotype by activating PKM2 and thereby inhibiting glycolysis (Wang et al., 2022).

Under steady-state conditions, macrophages generate ATP through the TCA cycle and mitochondrial oxidative phosphorylation. Conversely, hypoxia inhibits the TCA cycle, making glycolysis the primary metabolic pathway responsible for converting glucose into usable energy (El Kasmi and Stenmark, 2015; Fuller and Kim, 2021). Hypoxia-inducible factor 1α(HIF-1α) acts as a switch promoting anaerobic glycolysis in hypoxic macrophages and leads to the expression of the glucose transporter 1 (GLUT1) gene (Kelly and O'Neill, 2015). In cells overexpressing GLUT1, coenzyme A deficiency may limit the influx of excess pyruvate or fatty acids into the TCA cycle for oxidative metabolism (Wang et al., 2019). Succinyl-CoA is a TCA cycle intermediate typically confined to the mitochondrial matrix. It serves as a substrate for SDH. Excess cytoplasmic succinate is known to stabilize HIF-1α by inhibiting prolyl hydroxylase (Chelakkot et al., 2023), forming a positive feedback loop that further intensifies glycolysis and promotes inflammatory polarization. In summary, the primary metabolic feature of macrophages under hypoxic conditions is a shift from oxidative phosphorylation to glycolysis, which further promotes macrophage inflammatory polarization.

### 2.2 TCA and oxidative phosphorylation

The TCA cycle is the core pathway of cellular metabolism, comprising the following key steps: Acetyl-CoA (2-carbon, derived from fatty acid, amino acid, or pyruvate metabolism) condenses with oxaloacetate (4-carbon) to form citrate (6-carbon) under the catalysis of citrate synthase. Citrate is then isomerized to isocitrate by isocitrate dehydrogenase. Isocitrate is converted to α-ketoglutarate by isocitrate dehydrogenase (IDH), then to succinyl-CoA by the α-ketoglutarate dehydrogenase complex, which is then converted to succinate. Succinate is catalyzed by SDH to form fumarate, which is successively converted by malate dehydrogenase to malate and by fumarate dehydrogenase to oxaloacetic acid. Oxaloacetic acid initiates the process, completing the cycle (Martínez-Reyes and Chandel, 2020). The TCA cycle yields two primary products: reduced nicotinamide adenine dinucleotide (NADH) and reduced flavin adenine dinucleotide (FADH_2_). These can transfer electrons to the electron transport chain (ETC) to support oxidative phosphorylation and efficient ATP production under aerobic conditions, referred to as OXPHOS (Ao-Di et al., 2024). Activated macrophages (commonly termed M2) primarily utilize oxidative phosphorylation to generate energy-rich molecules such as ATP, participating in tissue repair and the downregulation of inflammation (Russo et al., 2021).

### 2.3 Fatty acid oxidation and amino acid metabolism

Macrophage energy supply is highly dependent on the synergistic interaction between FAO and amino acid metabolism. Glutamine, as a key metabolic substrate, can generate ATP via the TCA cycle and also be converted into citrate to participate in fatty acid synthesis. The metabolism of arginine and tryptophan, via the iNOS (inducible nitric oxide synthase) and arginase-1 pathways respectively, regulates the immune effector functions of macrophage polarization subsets (classically activated M1/selectively activated M2) (Van den Bossche et al., 2017).

At the fatty acid metabolic level, oxidation involves a four-step cyclic reaction: dehydrogenation, hydration, re-dehydrogenation, and desulfurization. Ultimately, fatty acids are converted into acetyl-CoA within mitochondria and undergo complete oxidation via the TCA cycle, providing substantial cellular energy (O'Neill et al., 2016; Yan and Horng, 2020). This process, alongside amino acid metabolism, forms the core energy supply network of macrophages, ensuring their ability to respond to pathogens (e.g., M1 pro-inflammatory response) while maintaining tissue homeostasis (e.g., M2 anti-inflammatory repair).

## 3 Macrophage polarization and immunemetabolism in gouty arthritis

As central effector cells of the innate immune system, macrophages play a critical role in defending against infection and maintaining tissue homeostasis (Ao-Di et al., 2024). Depending on differences in microenvironmental stimuli, macrophages primarily polarize into pro-inflammatory M1 or anti-inflammatory/repair-oriented M2 types (Hou et al., 2023), with the M2 subset further subdivided into M2a, M2b, M2c, and M2d subtypes. This is also the standard paradigm for studying macrophage function *in vitro* (Gadian, 1983). M1 macrophages are characterized by anti-pathogenic activity and a pro-inflammatory phenotype, whereas M2 macrophages dominate anti-inflammatory responses and tissue repair processes (Italiani and Boraschi, 2014). Under the induction of lipopolysaccharide (LPS) and interferon-γ (IFN-γ), classically activated M1 macrophages exhibit potent phagocytic capacity and secrete large amounts of pro-inflammatory cytokines (such as IL-1β, IL-6, TNF-α) and chemokines (such as CXCL9/10/11) to maintain a local pro-inflammatory microenvironment. In contrast, M2 macrophages, which are activated by alternative pathways involving anti-inflammatory factors such as IL-4, stimulate fibroblasts by secreting IL-10, CCL17, and various growth factors (e.g., VEGF, TGF-β), thereby promoting angiogenesis and extracellular matrix deposition, and thus play a leading role in tissue remodeling and repair processes (Sica et al., 2015; Geissmann et al., 2003; Hanna et al., 2011). Recent studies have shown that the functional phenotype of macrophages is highly correlated with their underlying cellular metabolic characteristics (Kopecky and Lavine, 2024). M1 macrophages undergo significant metabolic reprogramming, prioritizing aerobic glycolysis over mitochondrial OXPHOS for energy production. This metabolic shift not only rapidly provides the energy and biosynthetic substrates required for cell activation but is also accompanied by the release of large amounts of ROS,while this is crucial for clearing pathogens, it simultaneously induces oxidative stress and exacerbates local inflammation (Zhao W. et al., 2022). In contrast, M2 macrophages primarily rely on OXPHOS for energy to support their sustained anti-inflammatory and oxidative damage-suppressing functions (Ao-Di et al., 2024). Therefore, altering the metabolic profile of macrophages is a key mechanism driving their transition between pro-inflammatory and reparative phenotypes. During the onset and progression of gout, M1/M2 macrophages exhibit a characteristic dynamic evolution. In the early stages of gout, the numbers of local M1 and M2 macrophages are roughly equal; however, with the onset of acute gouty inflammation, M1 macrophages rapidly proliferate and become the dominant population, reaching a peak in number when inflammation peaks, accompanied by a decline in M2 macrophages (Gadian, 1983). Subsequently, as the disease transitions to the remission or chronic phase, changes in the local microenvironment lead to a gradual decrease in M1 numbers and a continuous rise in M2 numbers, ultimately resulting in the M2 macrophage population outnumbering the M1 macrophage population (Zhao L. et al., 2022). In summary, the dynamic imbalance in M1/M2 macrophage polarization within the gout microenvironment is a key determinant of disease progression.

The activation of immune cells is accompanied by significant metabolic reprogramming. This process not only reshapes the cell’s bioenergetic production patterns but also directly determines its immune effector functions. The precise coupling between these metabolic pathways and immune responses forms the core of the field of “immunometabolism”, regarded as a major conceptual breakthrough in modern immunology (Shan et al., 2025). The TCA cycle serves as the central metabolic hub in most mammalian cells and undergoes characteristic metabolic reprogramming in activated macrophages. Specific disruptions in this cycle lead to the accumulation of key metabolic intermediates, which have now been demonstrated to possess non-metabolic functions as immune signaling molecules (Peace and O'Neill, 2022).

The phenotypic and functional changes in macrophages are accompanied by significant alterations in cellular metabolism. Notably, M1 macrophages primarily rely on glycolysis, exhibiting two breaks in the TCA cycle that lead to the accumulation of itaconic acid (a bactericidal compound) and succinate. Excess succinate stabilizes HIF-1α, which activates transcription of glycolytic genes, thereby sustaining glycolytic metabolism in M1 macrophages. Conversely, M2 cells rely more heavily on OXPHOS, maintain an intact TCA cycle, and supply substrates to the electron transport chain (ETC) complexes (Viola et al., 2019).

The reprogramming of immune cell metabolic pathways and processes has become a crucial component of immune responses. Metabolic intermediates accumulate due to metabolic adaptation and exert functions beyond metabolism in immune and inflammatory regulation (Pålsson-McDermott and O'Neill, 2025).

In LPS-activated macrophages, the TCA cycle and OXPHOS are initially upregulated, followed by nitric oxide-mediated disruption of the Krebs cycle and respiratory chain, leading to accumulation of immune metabolites such as succinate, fumarate, and itaconic acid. These immunometabolites possess diverse immunoregulatory functions (Peace and O'Neill, 2022).

Itaconate is a key metabolite generated by cis-aconitase (ACOD1) and primarily secreted into the extracellular microenvironment by activated macrophages. Beyond its complex biological activities, itaconate is recognized as a potential therapeutic candidate for various inflammatory diseases due to its significant anti-inflammatory potential (Fan et al., 2025). Beyond meeting energy and biosynthetic demands, metabolites also function as signaling molecules. Under both infectious and non-infectious inflammatory conditions, itaconic acid (ITA) rapidly accumulates to high levels in myeloid cells. This metabolite binds to and modulates the function of multiple intracellular proteins, thereby influencing metabolism, oxidative stress, epigenetic modifications, and gene expression. It also signals extracellularly by binding to G protein-coupled receptors (GPCRs) (Ye et al., 2024). The immunomodulatory metabolite itaconic acid accumulates in innate immune cells following Toll-like receptor stimulation. After lipopolysaccharide activates macrophages, itaconic acid inhibits inflammasome activation and enhances type I interferon signaling (Paulenda et al., 2025). Concurrently, itexanic acid exerts immunomodulatory effects through multidimensional mechanisms: It not only regulates levels of the inflammatory metabolite succinate by inhibiting SDH, but also suppresses glycolysis at multiple nodes to limit inflammatory flux, while activating anti-inflammatory transcription factors (e.g., Nrf2 and ATF3) and inhibiting NLRP3 inflammasome assembly. Given these mechanisms, itaconic acid and its derivatives have demonstrated significant anti-inflammatory potential in preclinical models of diverse diseases including gout, sepsis, viral infections, psoriasis, ischemia/reperfusion injury, and pulmonary fibrosis (Peace and O'Neill, 2022). Furthermore, itaconic acid and its derivatives, metabolites derived from the TCA cycle, can suppress inflammatory responses in pro-inflammatory M1 macrophages (Runtsch et al., 2022).

Dysfunction of the TCA cycle leads to abnormal accumulation of succinate in the mitochondrial matrix, followed by its transmembrane transport into the cytoplasm and subsequent export to the extracellular space (Huang et al., 2024). As a classic TCA cycle metabolite (Trauelsen et al., 2021), abnormal physiological concentrations of succinate have emerged as a research focus in relation to inflammatory diseases triggered by excessive immune cell responses (Wei et al., 2023). Succinate serves both as a substrate for SDH and as an electron donor in the electron transport chain (ETC) (Martínez-Reyes and Chandel, 2020). Succinate is a crucial intermediate in mitochondrial energy metabolism. Recent studies indicate that beyond its known traditional metabolic functions, succinate also plays significant roles in signal transduction, immunity, inflammation, and post-translational modifications (Zhang S. et al., 2022). As a key metabolic signaling molecule, succinate drives inflammatory responses through dual mechanisms: intracellularly, high succinate concentrations stabilize HIF-1α by competitively inhibiting prolyl hydroxylases (PHDs), thereby initiating proinflammatory transcriptional programs. Extracellularly, it acts as a ligand binding to succinate receptor 1 (SUCNR1) on cell membranes to regulate effector functions of immune cells. This succinate-mediated immune hyperactivation has been demonstrated as a common metabolic basis for multiple pathological states including rheumatoid arthritis, inflammatory bowel disease, obesity, and atherosclerosis (Huang et al., 2024). The role of intracellular succinate as a pro-inflammatory mediator is gaining increasing attention (Murphy and O'Neill, 2018).

The inflammatory response in AGA is primarily triggered by the interaction between MSU crystals and macrophages. Upon stimulation by MSU, inflammatory macrophages undergo metabolic reprogramming, shifting their metabolic profile from glycolysis to oxidative phosphorylation. This shift in metabolic pathways further alters the function of pathogenic macrophages and ultimately drives the assembly and activation of the NLRP3 inflammasome (Li Z. et al., 2025; Ma et al., 2025). In LPS-activated macrophages, SDH catalyzes the oxidation of succinate to fumarate, a process that induces massive production of mitochondrial reactive oxygen species (mtROS), thereby driving the cell toward a pro-inflammatory metabolic phenotype. Conversely, targeted inhibition of SDH blocks the oxidative pathway of succinate, leading to its accumulation and effectively curbing the mtROS burst driven by mitochondrial complex I (Peace and O'Neill, 2022). Notably, succinate plays a dual role as a key metabolic signaling molecule: Intracellularly, due to its central role in the TCA cycle, it profoundly reshapes cellular metabolism by directly driving ROS production, stabilizing HIF-1α, and promoting protein succinylation; Extracellularly, succinate can also activate downstream signaling cascades by binding to its specific receptor, GPR91, thereby triggering a comprehensive and complex cellular response (Chen et al., 2024). As a transcriptional hub, stable and highly activated HIF-1α subsequently drives the expression of two classes of core targets: on the one hand, it transcriptionally activates glycolytic gene clusters such as GLUT1, LDHA, and MCT4 to maintain metabolic reprogramming; on the other hand, it induces pro-inflammatory factors such as iNOS, IL-1β, and IL-6 to amplify the immune response (Wu et al., 2024). A hallmark of macrophage polarization toward the M1 phenotype is enhanced glycolysis accompanied by disruption of the TCA cycle. This metabolic remodeling leads to the massive accumulation of key metabolic intermediates such as succinate, lactate, and L-2-hydroxyglutarate, which stabilize HIF-1α by inhibiting the activity of proline hydroxylase (PHD) (Tannahill et al., 2013; Feng et al., 2022; Williams et al., 2022). Concurrently, LPS promotes the dimerization and nuclear translocation of PKM2. Under inflammatory stress, PKM2 binds to the key transcription factor HIF-1α to form a pro-inflammatory complex, leading to the massive maturation and release of IL-1β (Palsson-McDermott et al., 2015). These mechanisms profoundly reveal the pivotal role of cellular metabolites in driving and maintaining inflammatory networks. Furthermore, the cascades linking metabolism and inflammation do not occur in isolation. As a byproduct of aerobic glycolysis, lactate also acts as a signaling molecule, mediating the lysine lactylation modification of proteins (Zhang W. et al., 2025). In particular, histone lactylation serves as a bridge linking cellular metabolism and gene transcription; it converts lactate signals generated by glycolysis into epigenetic regulation, thereby profoundly influencing the polarization process of macrophages (Chen et al., 2022; Li et al., 2024). Changes in the polarization state of macrophages are a key factor determining the clinical outcome of gouty arthritis (Song et al., 2025a).

Therefore, targeting metabolic hubs such as SDH or PKM2 not only blocks acute NLRP3 activation but also eliminates long-term inflammatory epigenetic memory by cutting off the supply of lactate.

During macrophage activation, their core metabolic pathways undergo a significant shift from oxidative phosphorylation to glycolysis. This metabolic shift leads to an impairment of the citric acid cycle, resulting in the massive accumulation of succinate, a mature pro-inflammatory metabolite (Tannahill et al., 2013). *In vitro* and *in vivo* studies have established a direct link between this metabolic disruption and the pathogenesis of arthritis: accumulated succinate not only activates macrophages via the specific receptor GPR91 (Littlewood-Evans et al., 2016), but also stabilizes HIF-1α, a key transcription factor regulating pro-inflammatory gene expression, ultimately driving the massive release of interleukin-1β (IL-1β) and directly exacerbating joint inflammation (Mills et al., 2016). In light of this, targeted intervention in the abnormal metabolism of macrophages has become a key strategy for reversing the progression of arthritis. Itaconic acid and its cell-permeable derivative 4-octyl itaconic acid (4-OI), as multifunctional immunomodulatory metabolites, exert distinct protective effects through three core mechanisms: stabilizing the immunosuppressive transcription factor Nrf2 (Nfe2l2), inhibiting glyceraldehyde-3-phosphate dehydrogenase (Gapdh) to block aerobic glycolysis, and competitively inhibiting SDH activity (Kachler et al., 2024). In terms of mechanism validation, 4-OI has demonstrated significant therapeutic efficacy in multiple animal models: in TNF transgenic/Irg1^−/−^ hybrid mice exhibiting severe bone destruction, 4-OI effectively alleviated synovial inflammation and bone destruction associated with rheumatoid arthritis (Rong et al., 2025). In a DMM-induced arthritis model, 4-OI promoted Nrf2 nuclear translocation, effectively reversed local apoptosis and matrix degradation, and protected articular cartilage (Zhang Q. et al., 2022). In a post-traumatic osteoarthritis model, 4-OI significantly inhibited the abnormal infiltration of pro-inflammatory M1 macrophages in the synovial endothelial layer, reduced synovial fibrosis, and comprehensively improved the synovial microenvironment at both imaging and histological levels while alleviating pain behavior (Tang et al., 2025).

Given that itaconic acid and its cell-permeable derivative, 4-OI have demonstrated clear therapeutic efficacy in animal models of rheumatoid arthritis, osteoarthritis, and post-traumatic osteoarthritis, this provides an important theoretical basis and translational approach for their future application in the treatment of GA.

**FIGURE 2:**
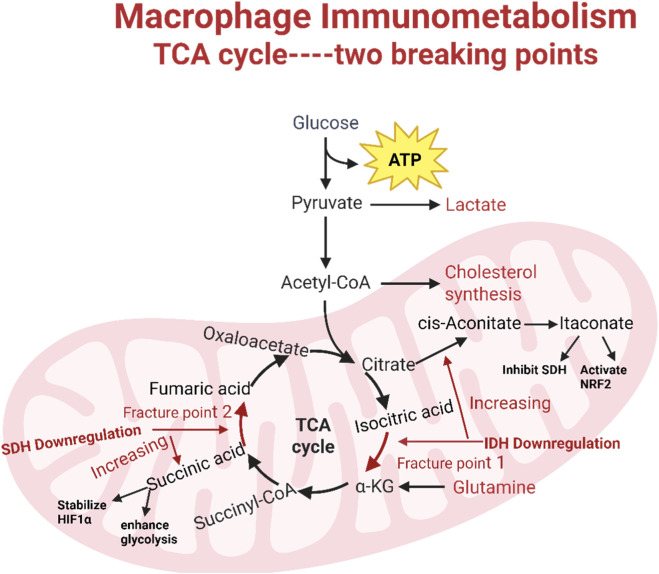
Macrophage immune metabolism, two cleavage sites, and the function of key metabolic intermediate products.

In summary, we have elaborated on the two “breakpoints” in macrophage polarization, immunometabolism and the TCA cycle, along with the functions of their metabolites in disease-related inflammation (see Figure 2). These findings not only deepen our understanding of macrophage immunometabolism but also clearly indicate that dysregulated TCA cycle and its metabolite signaling play a pivotal role in inflammation development. In the future, targeting the regulation of macrophage immune metabolism holds promise as an effective therapeutic strategy for such diseases.

## 4 Lactate and lactylation: the metabolic-epigenetic axis of GA

In the pathological process of GA, metabolic disorders within the local joint microenvironment and the dynamic regulation of macrophage energy metabolism form a complex bidirectional interaction network. During the acute inflammatory phase, MSU crystal deposition triggers tissue damage (Ahn and So, 2025), leading to the conversion of extracellular ATP into adenosine by nucleotidases (Toller-Kawahisa et al., 2025). Simultaneously regulating macrophage metabolic reprogramming. Enhanced glycolysis promotes excessive lactate accumulation (pH may drop below 6.5) (Xu B. et al., 2024), forming a characteristic acidic microenvironment. Lactic acid is primarily transported intracellularly and intercellularly via monocarboxylate transporters (MCTs), which facilitate its passage across the plasma membrane. Interestingly, this transport is synergistically influenced by pH, lactate concentration gradients, and cellular redox status (Sun et al., 2017; Ye et al., 2022). This low pH state impacts electron transport chain (ETC) efficiency by affecting the activity of mitochondrial complex I (NADH dehydrogenase), while AARS2-mediated mitochondrial protein lactylation inhibits acetyl-CoA production, directly suppressing mitochondrial OXPHOS and forcing macrophages to polarize toward a glycolytic metabolic phenotype (M1 type) (Okoye et al., 2023; Mao et al., 2024). M1 macrophage polarization produces inflammatory cytokines such as IL-6, IL-1β, TNF-α, and IL-12, promoting inflammatory progression (Fang et al., 2024). Simultaneously, lactate—the end product of fermentative glycolysis—can bidirectionally shuttle across the cell membrane via monocarboxylate transporters (MCT1/4). This facilitates cellular pH homeostasis, enhances HIF-1α stability, and induces high expression of glycolytic enzymes, forming a “metabolism-inflammation” positive feedback loop (Pouysségur et al., 2022).

Lactic acid, derived from pyruvate, is widely recognized as an energy source and metabolic byproduct, but emerging evidence suggests it functions as a critical signaling molecule. Studies indicate that histone lysine residue lactylation—a modification derived from lactic acid—constitutes an epigenetic modification that directly stimulates gene transcription on chromatin (Zhang et al., 2019). As a novel post-translational modification, histone lactylation directly links glycolytic metabolic flux to epigenetic regulation, forming a pivotal hub connecting metabolism and gene transcription (Li et al., 2024). Research indicates this modification serves as a vital cellular response mechanism under metabolic stress conditions (Qiao et al., 2024). Further studies confirm that protein lactylation is essential for lactate function, participating in critical biological processes such as glycolysis-related cellular functions and macrophage polarization (Chen et al., 2022).

Epigenetic modifications have been identified as key molecular determinants influencing macrophage plasticity and heterogeneity. Increasing evidence indicates that lactate-mediated alterations in histone lactylation levels within macrophages can regulate gene transcription, thereby participating in the pathogenesis of various diseases (Bao et al., 2025). Histone lactylation (particularly H3K18la) and lactate jointly regulate pathways involving inflammatory mediators, such as chemokine signaling and transient receptor potential channels. Reduced H3K18la levels alleviate symptoms in collagen-induced arthritis (CIA) models and mitigate the pathogenic effects of TNF-α on fibroblast-like synovial cells (FLS), whereas elevated H3K18la levels exacerbate TNF-α-induced pathological damage. Other studies indicate a positive feedback loop between the H3K18la target gene TTK/BUB1B and glycolysis/lactate production. Knockdown of TTK and BUB1B reduces histone lactate synthase (P300) expression, while TTK knockdown inhibits LDHA phosphorylation at site Y239. This leads to decreased lactate production and histone lactate levels, thereby affecting the expression of glycolysis-related genes (such as TTK itself) (Li et al., 2024). This represents a “metabolic-epigenetic” positive feedback vicious cycle. Histone deacetylases 1 (HDAC1) and 3 (HDAC3) exhibit site-specific deacetylase activity in cells, reducing H3K18 and H4K5 acetylation. Notably, HDAC3 knockout significantly increased H4K5 acetylation, indicating that HDAC1 and HDAC3 effectively function as “erasers” for histone acetylation modifications (Moreno-Yruela et al., 2022).

Additionally, a feedback loop exists between histone H4K12 lactylation (H4K12la) and HDAC3 in macrophages. Excessive H4K12la lactylation suppresses HDAC3 expression, while HDAC3 activation reduces H4K12la levels in macrophages (Zou et al., 2025).

**FIGURE 3:**
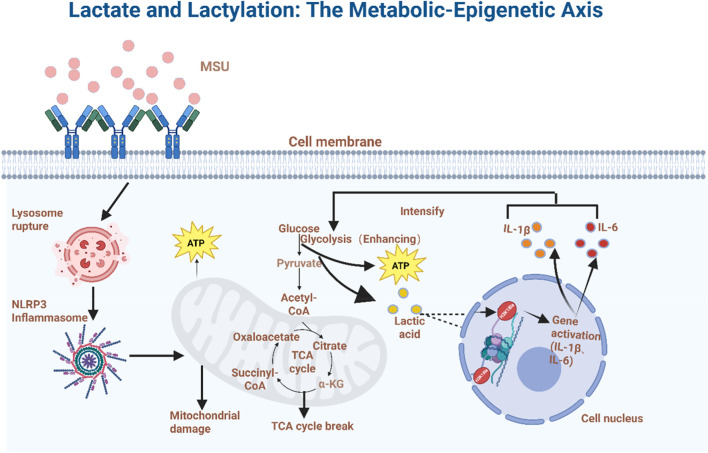
Lactate and lactylation: the metabolic-epigenetic axis of GA.

Through understanding the metabolic-epigenetic axis formed by lactate and lactation modification in GA, we discovered that regulating lactate levels or intervening in lactation modification holds promise as a novel therapeutic approach for GA (See Figure 3). Given this, targeted metabolic-epigenetic therapies represent a key future development direction for treating GA.

## 5 Therapeutic strategies targeting macrophage metabolism

GA is a chronic progressive disease characterized by elevated intra-articular urate levels and a persistent inflammatory immune microenvironment. However, clinical practice demonstrates that urate-lowering or anti-inflammatory therapies alone often fail to achieve satisfactory outcomes (Xu J. et al., 2024). This underscores the necessity for developing novel therapeutic strategies. Emerging research indicates that the immunometabolic state of macrophages is closely linked to their phenotype and function. Pro-inflammatory M1 macrophages primarily rely on glycolysis for energy, with impaired TCA cycle and mitochondrial OXPHOS. Conversely, anti-inflammatory M2 macrophages predominantly obtain energy through OXPHOS. Therefore, modifying the metabolic profile of macrophages can directly regulate their phenotype and function (Ao-Di et al., 2024). Based on this, targeting specific metabolic checkpoints—such as inhibiting rate-limiting enzymes like HK2 or blocking succinate-driven HIF-1α stabilization—has become a precise strategy to disrupt the energetic foundation of NLRP3 inflammasome activation in GA.

Currently, novel therapeutic approaches for GA are increasingly emerging, primarily encompassing the following aspects: restoring gut-immune homeostasis, modulating glycolysis and mitochondrial dynamics, intervening in signaling pathways, targeting epigenetic modifications and signaling molecules, and utilizing smart nanodelivery systems. To objectively assess the clinical translation potential of these strategies, we conducted a systematic comparative analysis of their core metabolic targets, key experimental endpoints, and current limitations, as presented in Table 1.

**TABLE 1:** Mechanism-driven therapeutic strategies.

Classification of intervention strategies	Representative intervention measures	Key metabolic/mechanistic targets	Comparison of key experimental parameters	References
Gut-immune homeostasis	*Clostridium* butyricum	Butyrate-mediated metabolic reprogramming	Intestinal microbiota diversity ↑	Song et al. (2025a)
	QZTBD	Butyrate-mediated metabolic reprogramming	ECAR ↓, OCR ↑	Song et al. (2025b)
Glycolysis and mitochondrial dynamics	GSE	Arg/iNOS shunt	The ECAR/OCR ratio reversed	Zhao W. et al. (2022)
	FHME	Mitochondrial morphological protection	Restoration of mitochondrial membrane potential	Zhao et al. (2025)
	Simiao Wan	Regulation of JAK2/STAT3	iNOS ↓,Arg-1 ↑	Yang et al. (2021)
Signal pathway intervention	Sirt1	Inhibits MAPK/NF-κB/AP-1 and activates Nrf2/HO-1	The expressions of IL-1β, TNF-α proteins and mRNAs ↓, Lcp1 ↑	Zhao et al. (2024)
	TPL	Inhibit PI3K/Akt		Du et al. (2024)
	RV	Inhibition of HIF-1α and the NLRP3 inflammasome		Wang et al. (2023)
	Tetrandrine	Inhibit NF-κB activity		Fang et al. (2024)
	Tripterine	Regulates the miR-449a/NLRP3 axis and the STAT3/NF-κB pathway, inhibits the NLRP3 inflammasome		Wang (2021)
	SIN	Modulation of the NLRP3/IL-1β pathway		Zhang S. et al. (2025)
	Stefin B	Inhibiting the NLRP3 inflammasome		Lin et al. (2024)
Epigenetic modifications	miR-486-5p/HDAC3 inhibitor	FOXO1 degradation/STAT3 inhibition	Target gene luciferase activity ↓; Changes in metabolic-related transcripts	Hu et al. (2022) Yang et al. (2024)
Smart nanodelivery	EM2 bionic membrane/CMCS@SAG	Multimodal metabolic regulation of uric acid degradation	Local ROS fluorescence at the joint ↓, MSU crystal deposition ↓	Xu J. et al. (2024), Chen et al. (2025)

### 5.1 Restoring gut-immune homeostasis

Research by Siyue Song et al. has thoroughly investigated the therapeutic effects of *Clostridium* butyricum and butyrate on GA. Findings indicate that supplementation with *Clostridium* butyricum or butyrate effectively alleviates gout by restoring intestinal homeostasis, regulating miR-146a expression, and promoting macrophage polarization toward the anti-inflammatory M2 phenotype. Mechanistic analysis revealed that the SOCS7/JAK2-STAT3 signaling pathway serves as a key mediator for miR-146a-induced macrophage polarization skewing. Notably, the absence of butyrate-producing bacteria leads to reduced butyrate levels, triggering abnormal miR-146a expression. This abnormality constitutes a critical factor in macrophage polarization imbalance and GA flare-ups (Song et al., 2025b).

Another study revealed the mechanism by which Qu-zhuo-tong-bi decoction (QZTBD) alleviates GA. QZTBD effectively elevated systemic butyrate levels by enriching butyrate-producing bacteria in the gut. At the mechanistic level, bone marrow-derived macrophages (BMDMs) treated with QZTBD serum or butyrate exhibited reduced extracellular acidification rate (ECAR) and oxygen consumption rate (OCR), indicating inhibition of glycolysis and restoration of mitochondrial OXPHOS. This metabolic shift significantly impedes pro-inflammatory M1 macrophage differentiation while promoting anti-inflammatory M2 macrophage polarization, ultimately achieving remission of GA (Song et al., 2025b).

However, as we move toward clinical applications of the “gut-metabolite-immune” axis, we must critically acknowledge the limitations of existing research. First, there is a significant species gap between rodents and humans in terms of gut microbiota composition; probiotic interventions effective in mouse models may not achieve equivalent colonization in patients with gout. Second, short-chain fatty acids (such as butyrate) have an extremely short half-life in the body and low systemic bioavailability. There remains a lack of rigorous comparative pharmacokinetic (PK/PD) data and validation regarding how these compounds can cross the intestinal barrier and achieve stable, therapeutically effective concentrations in the synovial microenvironment of joints—concentrations sufficient to induce metabolic reprogramming of macrophages.

### 5.2 Regulating glycolysis and mitochondrial dynamics

Grossamide (GSE), derived from Polygonum multiflorum, achieves profound metabolic reprogramming by precisely regulating the arginine metabolic pathway. In pro-inflammatory microenvironments, excessive nitric oxide (NO) severely inhibits the mitochondrial respiratory chain. GSE effectively diverts arginine metabolism by upregulating arginase and downregulating iNOS, thereby lifting the inhibition of mitochondrial OXPHOS caused by NO. Metabolic flux analysis confirmed that GSE intervention successfully reduced the extracellular acidification rate (ECAR), a marker of glycolytic activity, and significantly increased the oxygen consumption rate (OCR), restoring intracellular redox (NAD+/NADH) and energy (ATP/ADP) homeostasis. This underlying metabolic correction directly deprives M1 macrophages of their metabolic foundation, thereby driving their polarization toward the tissue-repairing M2 phenotype (Zhao W. et al., 2022).

In terms of mitochondrial dynamics regulation, hibiscus leaf extract (FHME) exhibits significant mitochondrial protective mechanisms. Under the acute stress of GA, macrophage mitochondria are highly susceptible to morphological fragmentation and functional impairment, forcing the cells to rely on glycolysis. Intervention with FHME effectively maintains mitochondrial structural integrity and strongly stimulates the expression of key OXPHOS proteins. By protecting mitochondrial morphology and respiratory function, FHME successfully shifts the core of macrophage energy metabolism from glycolysis back to mitochondrial OXPHOS, This reversal of the metabolic pattern curbs M1 polarization and the massive release of IL-1β at the source, significantly alleviating local inflammatory responses (Zhao et al., 2025).

Furthermore, the therapeutic effects of classical Chinese herbal formulas can also be attributed to the redirection of metabolic enzymes. For example, Simiao Wan, which is widely used in the clinical treatment of GA, can induce a “switch” in the core metabolic enzymes of macrophages within the synovial microenvironment by regulating the JAK2/STAT3 signaling pathway. Simiao Wan significantly inhibits the expression of iNOS, which catalyzes NO production, while substantially enhancing the activity of Arg-1 (arginase-1). This enzymatic intervention not only blocks the production of pro-inflammatory metabolites but also provides metabolic substrates for the synthesis of polyamines and proline, thereby powerfully driving M2 macrophage-mediated inflammation resolution and tissue repair at the metabolic level (Yang et al., 2021).

In summary, these natural products and traditional Chinese medicine formulations share a highly consistent core therapeutic strategy: they forcibly induce the polarization of macrophages from pro-inflammatory M1 to anti-inflammatory M2 cells by inhibiting key metabolic enzymes, protecting mitochondrial function (maintaining membrane potential), or regulating specific signaling pathways (such as JAK/STAT3). This mechanism ultimately exerts anti-inflammatory effects and alleviates gout symptoms.

### 5.3 Interveneing in signaling pathways

The control of inflammation in GA largely depends on modulating macrophage polarization (shifting from pro-inflammatory M1 to anti-inflammatory M2) through signaling pathway interventions. Silent Information Regulator 1 (Sirt1) is a nicotinamide adenine dinucleotide-dependent deacetylase extensively studied in cell cycle, aging, and metabolic processes (Qiang et al., 2012; Li et al., 2013). Sirt1 modulates various physiological functions, including inflammation (Yang et al., 2022) and oxidative stress (Xue et al., 2023). Studies confirm that Sirt1 alleviates GA inflammation through multiple pathways. By inhibiting MAPK/NF-κB/AP-1 signaling while activating Nrf2/HO-1 pathways, Sirt1 reduces M1 polarization and promotes M2 polarization, thereby attenuating inflammatory responses in GA (Zhao et al., 2024). Additional studies indicate that Sirt1 modifies macrophage polarization through the PI3K/Akt/STAT6 pathway, thereby improving MSU-induced inflammation (Liu et al., 2019).

Multiple studies indicate that various natural compounds and traditional Chinese medicine formulations can alleviate GA inflammation by regulating macrophage polarization and NLRP3 inflammasome through targeting different signaling axes. Research reveals that Tripterygium wilfordii homofuranone (TPL) prevents and treats MSU crystal-induced AGA by modulating macrophage polarization and neutrophil activity via regulating the PI3K/Akt pathway (Du et al., 2024). Resveratrol (RV), a natural polyphenolic compound, exhibits effects comparable to colchicine in reducing ankle swelling and synovial inflammation in rats with acute GA. Its mechanism involves inhibiting HIF-1α and NLRP3 inflammasome-derived IL-1β secretion in macrophages, thereby alleviating MSU-induced GA (Wang et al., 2023).

Tetrandrine, a dibenzylisobenzorhizinine alkaloid, promotes M2 macrophage polarization and suppresses M1 macrophage polarization in AGA mouse models by inhibiting NF-κB activity and its mediated Lcp1 expression, thereby alleviating inflammation (Fang et al., 2024). The Total Saponin Fraction of Dioscorea Nipponica Makino exhibits significant anti-inflammatory effects in GA rat models by modulating M1/M2 macrophage polarization through the arachidonic acid signaling pathway (Zhou et al., 2023). Tripterine modulates macrophage polarization via the miR-449a/NLRP3 axis and STAT3/NF-κB pathway, suppressing NLRP3 inflammasome expression to alleviate GA symptoms (Wang, 2021). Sinomenine (SIN), the primary active component of Sinomenium, demonstrates efficacy in alleviating gout inflammatory symptoms in both *in vitro* and *in vivo* studies. Its mechanism may involve regulating the NLRP3/IL-1β pathway, M1 macrophage polarization, and NET formation, positioning it as a promising therapeutic agent for gout (Zhang S. et al., 2025).

Additionally, the interaction between neutrophil extracellular traps (NETs) and macrophages represents an emerging mechanism in GA. Studies indicate that peptidyl arginine deiminase 4 (PAD4)-dependent neutrophil extracellular trap (NET) formation is significantly associated with gouty inflammation. Research has revealed that MSU crystal-induced NETs (potential drivers include free DNA and histone H3) promote M1 polarization and NLRP3 activation in macrophages by targeting HK-2 (Tan et al., 2024b). Consequently, targeting NET formation is considered a potential therapeutic strategy for preventing gout attacks.

Recent studies indicate that interventions involving other bioactive factors demonstrate promising efficacy in treating GA. In research examining the therapeutic effects of Stefin B on mouse GA and macrophage polarization, Stefin B alleviated GA by inducing M2 polarization and inhibiting NLRP3 inflammasome activation (Lin et al., 2024). *In vivo* and *in vitro* studies revealed that natural ACTH (adrenocorticotropic hormone) alleviates arthritis-induced swelling and inflammatory cell infiltration, improving acute GA inflammation, by regulating macrophage phagocytosis and polarization and influencing the transcription of related genes, without altering cortisol levels (Xu et al., 2022).

In summary, all these studies underscore a core conclusion: shifting the polarization state of macrophages from pro-inflammatory M1 to anti-inflammatory M2 represents a key therapeutic strategy for GA. This is primarily achieved by intervening in specific signaling pathways (such as Sirt1, PI3K/Akt, NF-κB, STAT3) and inhibiting NLRP3 inflammasome activation.

Although the aforementioned signaling pathway inhibitors demonstrate similar efficacy in terms of conventional inflammatory markers (such as cytokine concentrations and joint swelling), their limitations become apparent when viewed through the rigorous lens of “metabolic reprogramming.” Most upstream signaling networks (such as NF-κB or PI3K/Akt) exhibit high biological pleiotropy; broad inhibition of these pathways often lacks absolute selectivity for local inflammatory macrophages and is highly likely to disrupt the basal physiological metabolism of normal tissue cells, leading to systemic immunosuppression. Future evaluation of intervention strategies should not be limited to merely “reducing downstream inflammatory factors,” but should instead delve deeper to compare the specificity of different drugs regarding their binding affinity (IC50 values) for “core metabolic rate-limiting enzymes” (such as PFKFB3 and PKM2).

### 5.4 Targeting the epigenetic and signaling modulators

In the pathological microenvironment of GA, there is close crosstalk between epigenetic modifications and metabolic reprogramming of macrophages. Since the accumulation of abnormal metabolites often directly drives changes in the epigenetic landscape, targeting these upstream epigenetic and signaling regulators has emerged as a new strategy for intervening in pathological processes by correcting metabolic dysregulation.

MicroRNAs (miRNAs) exert precise regulation on metabolic gene networks at the post-transcriptional level. For example, microRNA-486-5p (miR-486-5p), which is significantly downregulated in GA cell models, has forkhead box protein O1 (FOXO1) as its key target. FOXO1 is not only an amplifier of inflammatory signals but also a key transcription factor regulating cellular glucose and lipid metabolism. Studies have shown that upregulating miR-486-5p can effectively target and degrade FOXO1, This intervention directly blocks FOXO1-mediated abnormal metabolic transcriptional programs, cutting off substrate supply to pro-inflammatory macrophages in their high-energy-consuming state, thereby significantly reducing the expression of inflammatory factors such as TNF-α, IL-8, and IL-1β at the metabolic source (Hu et al., 2022). Similarly, miR-192-5p has been shown to block the downstream receptor tyrosine kinase-dependent metabolic activation pathways (such as glycolysis-promoting signals) by specifically inhibiting the expression of the epidermal regulator (EREG) protein, thereby depriving M1 macrophages of their activation basis at the level of energy metabolism and significantly improving the local inflammatory response in GA (An and Yin, 2021).

In addition, the role of histone modifiers in reshaping the metabolic phenotype of macrophages has attracted significant attention. As a key link between epigenetics and metabolic immunity, HDAC3 directly influences the activation state of inflammatory metabolic pathways. Recent studies have confirmed that in MSU crystal-induced gout inflammation, specific knockout or inhibition of HDAC3 within macrophages can potently block the TLR2/4-driven IL-6/STAT3 signaling cascade. Given that STAT3 is a key transcriptional regulator of macrophage mitochondrial metabolism and HIF-1α-mediated glycolysis, inhibiting the HDAC3/STAT3 signaling axis effectively disrupts the essential pathway for MSU crystal-induced glycolytic burst in M1 macrophages, thereby alleviating the severity of gouty inflammation from both epigenetic and metabolic perspectives, suggesting that HDAC3 represents a highly promising therapeutic target for intervening in the immunometabolic imbalance associated with gout (Yang et al., 2024).

In summary, although research on epigenetic interventions for GA is still in its early stages, existing evidence has clearly demonstrated the central role of epigenetic regulatory networks—represented by miRNAs and histone deacetylases (such as HDAC3)—in reshaping the metabolic phenotype of macrophages and blocking inflammatory cascades. This not only broadens our understanding of the pathogenesis of gout but also lays a solid theoretical foundation for the future development of innovative gout drugs targeting both epigenetic and metabolic pathways.

### 5.5 Intelligent nano-delivery

With technological advancements, smart nanodelivery systems and macrophage metabolic reprogramming have emerged as two key therapeutic approaches for GA. The core pathology of GA involves MSU crystal deposition, which triggers excessive ROS production and pro-inflammatory M1 macrophage activation, thereby exacerbating synovial inflammation. Smart nanodelivery systems offer tremendous potential for efficient GA treatment by enhancing drug bioavailability, enabling targeted delivery, achieving sustained controlled release, and minimizing systemic side effects (Zhu et al., 2023). Stimuli-responsive nanoenzymes, in particular, have garnered significant attention for their ability to release drugs specifically within inflammatory environments (Zhang and Tong, 2023). A novel study proposes a multimodal therapeutic approach using targeted nanosystems for treating GA (Wang et al., 2023).

#### 5.5.1 ROS-responsive nanoenzymes

The local microenvironment during an acute gout attack is typically characterized by extremely high levels of oxidative stress, and the abundance of ROS further drives the polarization of macrophages toward the pro-inflammatory M1 phenotype. ROS-responsive nanocases are specifically designed to exploit this pathological feature. For example, Mohapatra et al. developed a bio-mineralized metal nanocase (FALNZ) that targets M1 macrophages. Intra-articular injection of FALNZ effectively scavenges local oxidative stress, directly suppressing the ROS burst induced by MSU crystals, thereby significantly reducing M1 macrophage levels and alleviating joint swelling and pathological damage at the source (Mohapatra et al., 2023). Similarly, the HTO-MnO nanoparticles designed by Sathiyamoorthy et al. not only demonstrated potent ROS-inhibitory capabilities but also successfully repolarized pro-inflammatory macrophages to the quiescent M0 state, highlighting the precise therapeutic potential of nanozymes in reshaping the inflammatory microenvironment (Sathiyamoorthy et al., 2025).

#### 5.5.2 Biomimicry of macrophage membrane carriers

By leveraging the natural “inflammatory homing” properties of the macrophage membrane, biomimetic carriers can achieve precise targeting of drugs to inflamed joints. Ma et al. ingeniously designed melatonin-loaded liposomes coated with macrophage membrane (MLT-MLP). This system not only enables the precise delivery of melatonin but also, through its core mechanism, powerfully drives metabolic reprogramming in pathological macrophages—reversing their energy dependence from aerobic glycolysis to mitochondrial OXPHOS—thereby directly inducing anti-inflammatory polarization and alleviating tissue damage in acute GA (Ma et al., 2025). Furthermore, the multimodal nanosystem (EM2) developed by Zhang Jingqing’s team has taken biomimetic design to new heights. EM2 fuses M2 macrophage membranes with exosomes and encapsulates uricase, platinum/polydopamine nanozymes, and resveratrol. This system successfully achieved the dual-targeted objectives of “local uric acid degradation” and “metabolic reprogramming-driven M2 polarization,” providing a novel, highly efficient, and low-immunogenic multimodal therapeutic strategy for GA (Xu J. et al., 2024).

#### 5.5.3 Metabolite-regulated delivery vehicle

These carriers are designed to sustainably release metabolic modulators at the site of inflammation to fine-tune intracellular energy metabolism networks. For example, the novel Gas6-loaded hydrogel microspheres (CMCS@SAG) developed for the treatment of acute GA exhibit excellent *in situ* sustained-release and pH-responsive properties. *In vitro* and *in vivo* studies have shown that CMCS@SAG can be specifically triggered by the inflammatory microenvironment; the released drug significantly enhances mitochondrial function and promotes M2 polarization by directly altering the underlying metabolic reprogramming pathways of macrophages, demonstrating strong potential for anti-inflammatory and tissue repair (Chen et al., 2025). It is worth noting that the carrier material itself may also exert unintended effects on immune metabolism. For example, studies have shown that even macrophages in a resting (M0) or anti-inflammatory (M2) state exhibit abnormally heightened glycolytic metabolism upon exposure to aluminum-based adjuvants (Danielsson et al., 2023). This finding suggests that metabolic reprogramming in macrophages is highly sensitive to microenvironmental factors, when designing delivery carriers for gout treatment in the future, it is essential to rigorously evaluate the immunostimulatory properties of matrix materials to avoid exacerbating inflammation due to metabolic interference caused by the materials themselves.

## 6 Summary and future perspectives

Summary: Macrophage metabolic reprogramming in GA has emerged as a prominent research focus in recent years. Substantial basic research evidence indicates that macrophage metabolic reprogramming can alter macrophage phenotypes to exert anti-inflammatory or pro-inflammatory effects, thereby influencing the progression and remission of GA. Unlike traditional approaches solely focused on uric acid reduction and anti-inflammatory effects through inflammatory factor suppression, this review incorporates macrophage immunometabolism, TCA cycle breakpoints, and metabolite functions. It emphasizes the “metabolism-epigenetics” regulatory network in GA and thoroughly explores therapeutic strategies targeting macrophage polarization and metabolism, providing direction for future in-depth studies on epigenetics in GA. Finally, this review represents the first systematic integration of “macrophage metabolic reprogramming” and “smart nanomedicine targeting” in GA, offering a targeted approach that selectively delivers metabolic modulators (e.g., SDH inhibitors) to the inflamed synovium, mitigating systemic immunosuppression. To facilitate clinical application, future efforts must overcome the translational gap by utilizing humanized 3D synovial organoids to accurately validate metabolic fluxes prior to *in vivo* trials.

Outlook: A comprehensive evaluation of existing intervention strategies reveals that current research still has significant limitations. Existing *in vitro* studies rely heavily on stimulation with either LPS or MSU crystals alone, while *in vivo* experiments primarily rely on acute modeling methods. These models struggle to accurately replicate the chronic, recurrent hypoxia and high-fat metabolic disorders that persist in the joint cavities of human gout patients over the long term. Furthermore, most current literature uses ECAR and OCR solely as metabolic endpoints, with a severe lack of precise analysis of specific metabolite fluxes using carbon tracing or spatial metabolomics techniques. Although targeting macrophage metabolism has shown great therapeutic promise in preclinical animal models, bridging the “bench-to-bedside” gap between basic research and clinical application remains fraught with significant challenges: 1. The ubiquity of metabolic targets and off-target toxicity: Glycolysis and oxidative phosphorylation are fundamental pathways for sustaining cellular life throughout the body. Systemic use of non-selective metabolic inhibitors may cause irreversible toxic side effects in organs with high metabolic demands, such as the heart and brain. 2. High plasticity and temporal dynamics of macrophages: The M1/M2 transition in the inflammatory microenvironment is a highly dynamic process. Inappropriate metabolic inhibition may disrupt immune homeostasis and weaken the body’s normal defense against pathogens. 3. Limitations of Animal Models and Disease Heterogeneity: Existing animal models of acute gout often fail to fully replicate the true pathological microenvironment of human chronic, recurrent gout and the complex metabolic syndrome associated with it. To overcome the aforementioned translational bottlenecks and achieve truly precise, targeted therapy, future research urgently needs to delve deeper into the following areas: 1. Decoding more refined metabolic-epigenetic networks: Future research should expand its scope from glycolysis to fatty acid oxidation and amino acid metabolism; conduct in-depth analyses of how specific metabolites act as signaling molecules to dynamically interact with epigenetic modifications (such as histone acetylation and methylation), thereby identifying safer and more specific upstream intervention targets. 2. Characterize the spatiotemporal heterogeneity of macrophages: Macrophages within gouty joints have diverse origins (tissue-resident vs. monocyte-derived). Future research will leverage cutting-edge multi-omics technologies, such as single-cell RNA sequencing (scRNA-seq), spatial transcriptomics, and spatial metabolomics, to precisely map the dynamic metabolic profiles of different subpopulations across various stages of gout, with the aim of identifying “next-generation metabolic checkpoints” specific to pathological macrophages. 3. Constructing biomimetic models to investigate intercellular metabolic interactions: Macrophages do not exist in isolation. In the future, we should actively incorporate advanced *in vitro* 3D models, such as the “Synovium-on-a-Chip,” to observe and analyze in real time how macrophages, neutrophils, synovial fibroblasts, and T cells engage in immunometabolic crosstalk through the exchange of metabolites (e.g., lactate, amino acids). 4. Optimizing Smart Nanotargeted Delivery Technologies: Leveraging advances in materials science, develop smart nanocarriers responsive to the inflammatory microenvironment of gout (e.g., pH and ROS responsiveness). By precisely delivering metabolic modulators into target cells, systemic off-target toxicity can be minimized to the greatest extent possible, this is also the most critical step in advancing metabolic targeted therapies toward clinical application. While we acknowledge the current translational limitations—particularly regarding model fidelity and off-target effects—overcoming these hurdles remains the focal point of ongoing research. Ultimately, the seamless integration of metabolic-epigenetic insights with intelligent nanodelivery systems promises a profound improvement in the clinical management of gouty arthritis, driving a vital transition from temporary symptom relief to lasting disease resolution.

The broader scientific impact of this review lies in its unprecedented integration of macrophage immunometabolism with cutting-edge nanotherapeutics. By establishing the “metabolic-epigenetic”axis as a highly druggable target, this framework shifts the research paradigm of gouty arthritis from conventional symptom management toward targeted disease resolution, paving the way for the next-generation of precision nanomedicines.
